# Practical Departments

**Published:** 1904-12-10

**Authors:** 


### PRACTICAL DEPARTMENTS.

### THE "CRITON" WATER SOFTENER.

WE have received a *brochure* from the Pulsometer Engineering Company, of Nine Elms Works, Reading, describing the above apparatus. As our readers are aware, water is what is known as hard because it has dissolved certain salts, usually in its passage over rocks containing them, and the hardness has two possible inconveniences; it uses up soap wastefully when used for washing, and it causes a deposit of the salts on the inner surfaces of boilers when it is used for generating steam. In both cases the hardness of the water causes the users to incur more expense, often to a considerable amount, than where the hardness does not exist. In laundry work a large portion of the soap is used up in neutralising the salts which cause the hardness, before the proper work of the soap, the cleansing of the clothes, can be entered upon. In steam boilers the deposit forms a scale upon the surface of the boiler tubes, or other portions in contact with the water, reducing the available water space, and, what is of far more importance, interposing resistance in the path of the heat in its passage from the flue-gasses to the water. So great is this latter trouble that it is stated that, with a scale three-quarters of an inch thick, the heating efficiency is reduced by 90 per cent. In water-pipes also the trouble arises in another form. The deposit gradually fills up the pipes, obliging the water to be forced through them at continually increasing velocities, and to a very large increase of power being required to drive it through.

#### DEGREES OF HARDNESS.

It is usual to refer to water as having so many degrees of hardness. The term, though used in the same way as are degrees on a temperature scale, does not mean the same thing. It is an arbitrary scale, based upon the quantity of soap decomposed by one grain of carbonate of calcium. Thus, each degree of hardness means that each gallon of the water referred to contains that number of grains of carbonate of calcium in solution. A water with 5° of hardness would have 5 grains per gallon, and so on. The salts which cause the hardness of water are, fortunately, few in number, and they are divided into two groups, those which are deposited when the water is boiled, and those which do not. The former are the bicarbonates of calcium and magnesium. The metals calcium and magnesium combine with carbonic acid in two proportions — the carbonate and the bicarbonate—the former containing one chemical equivalent of carbonic acid, and the latter two equivalents. The carbonate is insoluble in water, while the bicarbonate is soluble. Hence, unless the bicarbonate is removed it becomes the carbonate in the boiler, and is deposited as described. The operation of getting rid of temporary hardness, as it is termed, getting rid really of the bicarbonates and the carbonates before the water enters the boiler, consists in boiling the water, or in adding to it a certain quantity of hydrate of lime, the result being that a portion of the carbonic acid is either driven off as a gas, or is absorbed by the lime. In either case the bicarbonates are reduced to carbonates, and deposited in a receptacle prepared for them before the water is allowed to pass into the boiler, or is used for washing or other purposes. The "Criton" apparatus adopts the latter method, which has the advantage that heating is not required, the whole operation being chemical and mechanical.

#### PERMANENT HARDNESS.

The salts which cause what is known as permanent hardness, because it is not removed by boiling, are principally the sulphate of calcium and the chloride of magnesium. The operation of removing these salts is purely chemical, with such mechanical arrangements as are necessary for continuous working. Carbonate of soda, when added to water containing sulphate of calcium, causes the sulphuric acid of the sulphate to pass from the lime to the soda, sulphate of sodium being formed in place of sulphate of lime. The chloride of magnesium is removed by the addition of caustic soda, sodium chloride being formed in place of magnesium chloride. As neither sodium sulphate nor sodium chloride are deposited in boilers, etc., nor have the properties associated with hardness, the conversion gets rid of the two salts, and of others, such as calcium chloride, of a similar nature. In the "Criton" apparatus the two operations for the removal of temporary hardness, and of permanent hardness are combined by adding to the water, in suitable proportions, carbonate of soda and hydrate of lime, the action of these two forming the caustic soda required for the magnesium chloride. In the apparatus there are a lime tank, a soda tank, both controlled by ball-cocks, and it is so arranged that the water passes into a mixing tank, with definite quantities of the two salts referred to, thence to a filter, and out to the service, the quantities of water flowing of carbonate of soda and of hydrate of lime passing into the mixing tank, being under the complete control of the operator.

